# Molecular Characterization of Carbonic Anhydrase Genes in *Lotus japonicus* and Their Potential Roles in Symbiotic Nitrogen Fixation

**DOI:** 10.3390/ijms22157766

**Published:** 2021-07-21

**Authors:** Longlong Wang, Jianjun Liang, Yu Zhou, Tao Tian, Baoli Zhang, Deqiang Duanmu

**Affiliations:** State Key Laboratory of Agricultural Microbiology, College of Life Science and Technology, Huazhong Agricultural University, Wuhan 430070, China; jianjunliang@webmail.hzau.edu.cn (J.L.); zhou_yu@webmail.hzau.edu.cn (Y.Z.); 2020304110157@webmail.hzau.edu.cn (T.T.); zhangbaoli123@webmail.hzau.edu.cn (B.Z.)

**Keywords:** carbonic anhydrase, *Lotus japonicus*, root nodule, symbiotic nitrogen fixation

## Abstract

Carbonic anhydrase (CA) plays a vital role in photosynthetic tissues of higher plants, whereas its non-photosynthetic role in the symbiotic root nodule was rarely characterized. In this study, 13 CA genes were identified in the model legume *Lotus japonicus* by comparison with Arabidopsis CA genes. Using qPCR and promoter-reporter fusion methods, three previously identified nodule-enhanced CA genes (*LjαCA2*, *LjαCA6*, and *LjβCA1*) have been further characterized, which exhibit different spatiotemporal expression patterns during nodule development. *LjαCA2* was expressed in the central infection zone of the mature nodule, including both infected and uninfected cells. *LjαCA6* was restricted to the vascular bundle of the root and nodule. As for *LjβCA1*, it was expressed in most cell types of nodule primordia but only in peripheral cortical cells and uninfected cells of the mature nodule. Using CRISPR/Cas9 technology, the knockout of *LjβCA1* or both *LjαCA2* and its homolog, *LjαCA1*, did not result in abnormal symbiotic phenotype compared with the wild-type plants, suggesting that LjβCA1 or LjαCA1/2 are not essential for the nitrogen fixation under normal symbiotic conditions. Nevertheless, the nodule-enhanced expression patterns and the diverse distributions in different types of cells imply their potential functions during root nodule symbiosis, such as CO_2_ fixation, N assimilation, and pH regulation, which await further investigations.

## 1. Introduction

Carbonic anhydrases (CAs) are ubiquitous in living organisms including prokaryotes, plants, and animals [1]. CAs are among the most efficient enzymes, which mostly contain a zinc ligand and catalyze the reversible hydration of carbon dioxide: CO_2_ + H_2_O ⇌ HCO_3_^−^ + H^+^ [2]. Higher plants contain three different types of CAs, namely αCA, βCA, and γCA. Each type of CA has multiple functional isoforms, which are widely expressed in photosynthetic or non-photosynthetic tissues and located in a variety of cellular compartments such as cytoplasm, plasma membrane, chloroplast, and mitochondria [3,4,5]. Various isoforms and diversified intracellular localizations of CAs correspond to the multiple biological functions of these enzymes in plants.

Three CA families have independent evolutionary history and likely have developed different biological functions. Although large numbers of αCA genes have been identified in plants, little information has been reported for their functions, intracellular locations, and expression patterns [3]. In contrast, γCAs are expressed in almost all tissues and play conserved roles in mitochondrial complex I, which indicates their housekeeping functions in maintaining mitochondrial function among different plant species [3,6]. βCAs are the most intensively studied CA family in plants. They were proposed to participate in facilitating the diffusion of CO_2_ across the chloroplast membranes and supply RuBisCO with CO_2_ in C3 plants [7]. They are also involved in the CO_2_ concentrating mechanism (CCM) in C4 plants and supply phosphoenolpyruvate carboxylase (PEPC) with HCO_3_^−^ [8]. Additionally, βCAs were found to regulate CO_2_-controlled stomatal development and movement in Arabidopsis and rice [9,10,11]. Recently, several non-photosynthetic functions of βCAs have been identified, including the regulation of intracellular pH and cell differentiation in the tapetal cells [12] and the activation of plant basal immunity [13]. Although well proposed, non-photosynthetic functions of βCAs still require further investigations, such as amino acid biosynthesis, lipid biosynthesis, and CO_2_ fixation in the dark [3].

Legume root nodules exhibit relatively high CA activity, implying the non-photosynthetic role of CA in symbiotic nitrogen fixation (SNF) [14]. *MsCA1*, the first CA gene cloned from non-photosynthetic tissue in plants, was expressed in all cell types of nodule primordia but exclusively in the peripheral cortical cells of mature nodules [15]. Notably, the expression level of *MsCA1* showed an inverse relationship with ambient O_2_ concentration, implying its potential role in the gas exchange of root nodules [16]. *GmCA1* and *LjCA1*, two homologous genes of *MsCA1* identified in *Glycine max* and *Lotus japonicus* respectively, showed similar expression patterns as *MsCA1* in the nitrogen-fixing nodule, implying their evolutionarily conserved functions inside symbiotic root nodules [17,18,19]. Additionally, two α type CAs (LjCAA1 and LjCAA2) in *Lotus japonicus* and another α type CA (MlCAA1) in *Mesorhizobium loti* R7A were identified and proposed to play essential roles in root nodule symbiosis [20,21,22]. Nonetheless, the presence of multiple CA isoforms and the absence of corresponding genetic mutant materials greatly hindered the investigation of the biological function of CAs during SNF.

In this study, we firstly performed the genome-wide identification of candidate carbonic anhydrase genes in *Lotus japonicus*. Considering the specialized function of γCAs in mitochondrial complex I, here, we mainly focused on the characterization of αCAs and βCAs in non-photosynthetic root nodules. Three nodule-enhanced CA genes (*LjβCA1*, *LjαCA2*, and *LjαCA6*) were characterized and exhibited diverse expression patterns in different cell types of root nodules. To elucidate the biological functions of these candidate CA genes, CRISPR/Cas9-mediated gene knockout experiments were performed. Three *LjβCA1* mutants and two *LjαCA1/2* mutants were obtained and used for phenotypic comparisons. These results would contribute to the functional characterization of multiple CA isoforms in maintaining an efficient SNF during legume–rhizobia symbiosis.

## 2. Results

### 2.1. Identification and Phylogenetic Analysis of Carbonic Anhydrase Genes in Arabidopsis and Lotus

**Figure 1:**
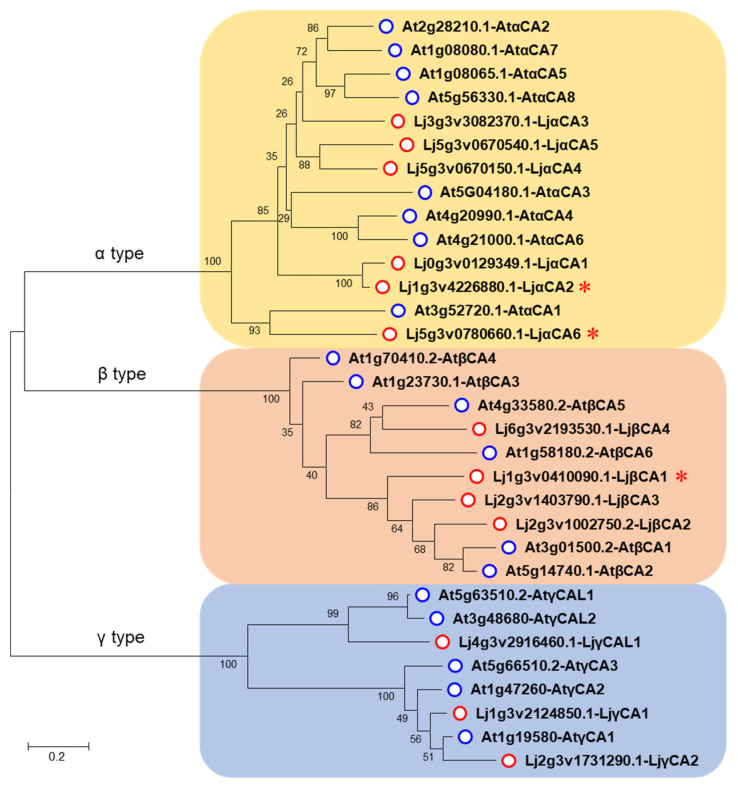
Phylogenetic tree of carbonic anhydrase genes in Lotus and Arabidopsis. The protein sequences were obtained from TAIR (http://www.arabidopsis.org/) (accessed on 19 September 2018) and Kazusa DNA Research Institute (http://www.kazusa.or.jp/lotus/) (accessed on 19 September 2018). Sequence alignment was performed using Clustal W, and the phylogenetic tree was generated by MEGA 7.0 with 1000 bootstrap replications. Blue circles indicate Arabidopsis CA proteins. Red circles indicate Lotus CA proteins. Red stars indicate three LjCAs with enhanced expression in root nodules. The scale bar indicates an evolutionary distance of 0.2 amino acid substitutions per position.

To identify the CA-encoding genes in the *Lotus japonicus* genome, we firstly performed a protein BLAST search using the Arabidopsis CA proteins as queries. Then, these protein sequences were examined by the NCBI Conserved Domain Database (CDD) to ensure that they contain the typical carbonic anhydrase domain. After removing the fragmentary and redundant sequences, a total of 13 putative CA genes were finally identified. To distinguish these candidates, these CA genes were subsequently labeled according to their sub-clade in CA phylogenetic tree and their chromosomal locations (Table 1, Figure 1). Notably, *LjαCA1* was not mapped to any chromosome in the Lotus V3.0 genome, and it has no introns or putative 5′ or 3′ untranslated regions (Figure S1A). Therefore, *LjαCA1* might be a pseudogene that was not regarded as the target for further analysis, although it shows above 95% homology with *LjαCA2* in protein-coding sequence (Figure S1B). Additionally, three previously identified Lotus CA genes were renamed in this study, including *LjCAA1* (renamed as *LjαCA2*), *LjCAA2* (*LjαCA6*), and *LjCA1* (*LjβCA1*) (Table 1).

To identify the evolutionary relationships among these CA genes, a neighbor-joining phylogenic tree was constructed according to protein sequences of Arabidopsis and Lotus enzymes. Similar to Arabidopsis, the Lotus genome encodes three types of CAs, including six α type CAs (LjαCA1–6), four β type CAs (LjβCA1–4), and three γ type CAs (LjγCA1–2, LjγCAL1) (Figure 1, Table 1). Physicochemical property analysis shows that the lengths of Lotus carbonic anhydrase proteins (LjCAs) range from 186 to 324 amino acids, the molecular weights of the LjCAs range from 20.2 to 34.9 kDa, and the isoelectric points range from 5.49 to 9.66. Notably, both basic and acidic proteins are present in α and γ type CAs, whereas all these four β type CAs are acidic. Additionally, the grand average of hydropathicity (GRAVY) values range from −0.058 to −0.639 except for LjγCAL1, indicating that most of the LjCAs are hydrophilic (Table 1).

### 2.2. Expression Profiles of LjCAs across Different Tissues and Different Developmental Stages of Root Nodule

**Figure 2:**
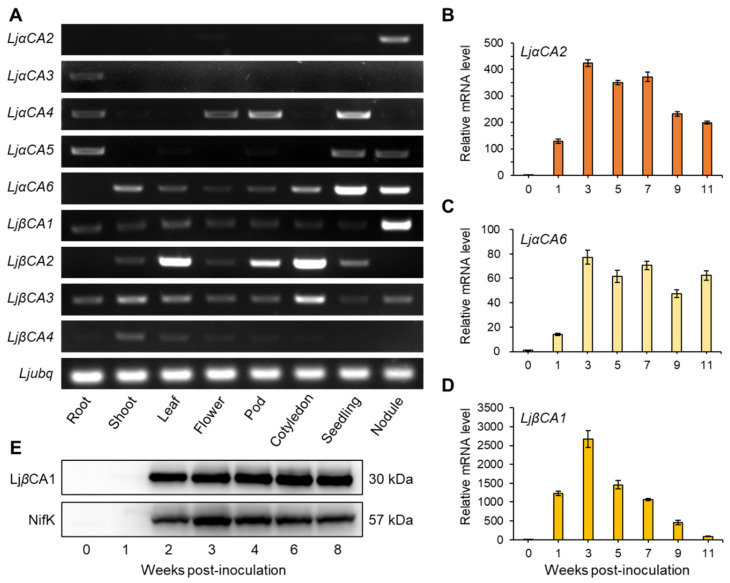
Expression patterns of carbonic anhydrase genes in *Lotus japonicus*. (**A**) RT-PCR analyses of α and β type CA genes in different tissues of *Lotus japonicus*. A total of 33 cycles were used for amplifying α type CA genes, while 29 cycles were used for amplifying the ubiquitin gene and β type CA genes. The ubiquitin gene was used as an internal control. Root, uninoculated root; Nodule, mature nodule at 3 wpi (weeks post-inoculation). (**B**–**D**) Expression profiles of *LjαCA2*, *LjαCA6*, and *LjβCA1* during nodule development. qRT-PCR was used to quantify the transcript abundance of *LjαCA2*, *LjαCA6*, and *LjβCA1* in the uninoculated root (0 wpi) and in developing nodule (1 to 11 wpi). Relative mRNA levels of three genes in 1 to 11 wpi with respect to 0 wpi were calculated using ubiquitin as a a reference gene. Values are means ± SD of three technical replications. Similar results were observed in three independent experiments. For (**A**–**D**), cDNA from 5 ng total RNA was used as the template for a 10 μL PCR reaction. (**E**) Representative immunoblot of LjβCA1 and NifK in uninfected root (0 wpi) and in developing nodule (1 to 8 wpi). The primary antibodies were polyclonal antibodies against LjβCA1 and NifK.

To identify the CA genes related to SNF, semi-quantitative RT-PCR was performed to investigate the expression profiles of *LjCAs* across different tissues, including root, shoot, leaf, flower, pod, cotyledon, seedling, and nodule. The results show that *LjαCA2*, *LjαCA6*, and *LjβCA1* are highly expressed in root nodules, indicating their potential roles in maintaining nodule function. *LjaCA6* demonstrates a higher expression level in seedlings, and it is also expressed in other tissues, including shoot and cotyledon. Hence, the biological function of *LjaCA6* may not be restricted to root nodules (Figure 2A). For other CA genes, they tend to express in non-symbiotic tissues, such as *LjαCA3* in root and *LjβCA2* in leaf, pod, and cotyledon. Additionally, *LjβCA3* is expressed in most tissues with a similar level, implying its potential housekeeping function (Figure 2A). The public expression profile data of *LjCAs* were also retrieved from *Lotus japonicus* Expression Atlas (https://lotus.au.dk/expat/) (accessed on 3 July 2021). As shown in Table S1, *LjαCA2*, *LjαCA6*, and *LjβCA1* are three dominant CA genes with enhanced expression in root nodule consistent with the RT-PCR results (Figure 2A).

To further investigate the expression patterns of three nodule-enhanced CA genes in detail, quantitative RT-PCR was performed to detect the transcript levels of *LjαCA2*, *LjαCA6*, and *LjβCA1* at different stages of nodule development. The transcript levels of these three genes are increased after rhizobial inoculation or during nodule maturation (Figure 2B–D). Among them, *LjαCA2* and *LjαCA6* exhibit remarkably high expression levels in mature nodules (3, 5, 7 wpi) and maintain the high expression levels in senescent nodules (9, 11 wpi, Figure 2B,C). However, the expression of *LjβCA1* is dramatically up-regulated at 3 wpi, reaching above 2,500-fold compared with that in uninoculated roots (0 wpi). After that, the expression level of *LjβCA1* gradually declines along with the development of root nodules (Figure 2D). Additionally, we also performed immunoblot for detecting LjβCA1 and NifK proteins at different nodule developmental stages. The LjβCA1 protein accumulates at 2 wpi; then, it maintains a high level at the later stages of nodule development (2 to 8 wpi). In contrast, the protein level of NifK peaks at 3 wpi and decreases after nodule maturation (Figure 2E). In summary, the nodule-enhanced expression patterns of *LjαCA2*, *LjαCA6*, and *LjβCA1* imply their potential functions in nodule maturation, nitrogen fixation, or even nodule senescence.

### 2.3. Spatiotemporal Expression Patterns of Nodule-Enhanced LjCAs in Root Nodule

**Figure 3:**
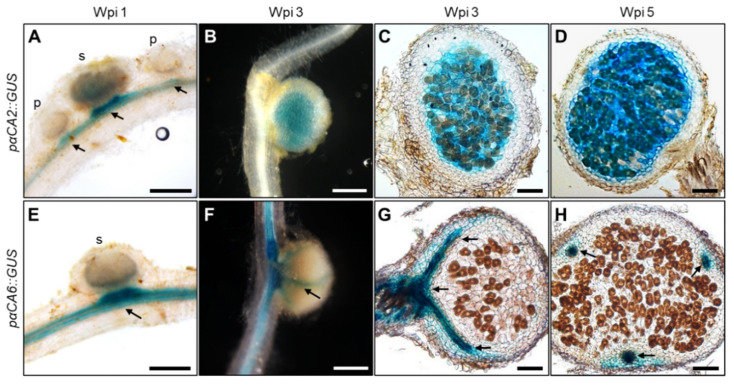
Histochemical analysis of GUS expressions driven by *LjαCA2* and *LjαCA6* promoters in developing root nodules. Expression patterns of GUS reporter were analyzed for *pαCA2::GUS* (**A**–**D**) and *pαCA6::GUS* (**E**–**H**). Nodules at different developmental stages were investigated, including young nodules at 1 wpi (**A**,**E**) and mature nodules at 3 wpi (**B**,**C**,**F**,**G**) and 5 wpi (**D**,**H**). *pαCA2::GUS* and *pαCA6::GUS* constructs were introduced into wild-type plants using stable transformation. T_2_ generation plants were used for GUS staining experiments. Images are representative of at least eight independent transgenic plants. Black arrows indicate the vascular bundle. p, primordia; s, small nodule. Scale bars, 200 μm (**A**,**E**); 1 mm (**B**,**F**); 100 μm (**C**,**D**,**G**,**H**).

We next performed promoter–reporter fusion experiments for further investigating the cell-specific expression patterns of *LjαCA2*, *LjαCA6*, and *LjβCA1*. Around 3 kb promoter regions of three CA genes were amplified and then fused to the GUS reporter gene. Stably transformed plants were generated and used for GUS staining experiments. As shown in Figure 3, the GUS staining signal of *pαCA2::GUS* transgenic lines is located in the vascular bundle of the root near the nodule at 1 wpi (week post-inoculation). No signal is detectable inside nodule primordia, while a weak signal is visible in small nodules (Figure 3A). During nodule maturation, *LjαCA2* is mainly expressed in the central nitrogen fixation zone, both in the infected and uninfected cells at 3 wpi (Figure 3B,C) and 5 wpi (Figure 3D). For *LjαCA6*, GUS staining signal is detectable in the root vascular bundle at 1 wpi (Figure 3E). In mature nodules, the expression of *LjαCA6* is limited to the nodule vascular bundle at 3 and 5 wpi, and no signal is detectable in the central nitrogen-fixing zone (Figure 3F–H).

**Figure 4:**
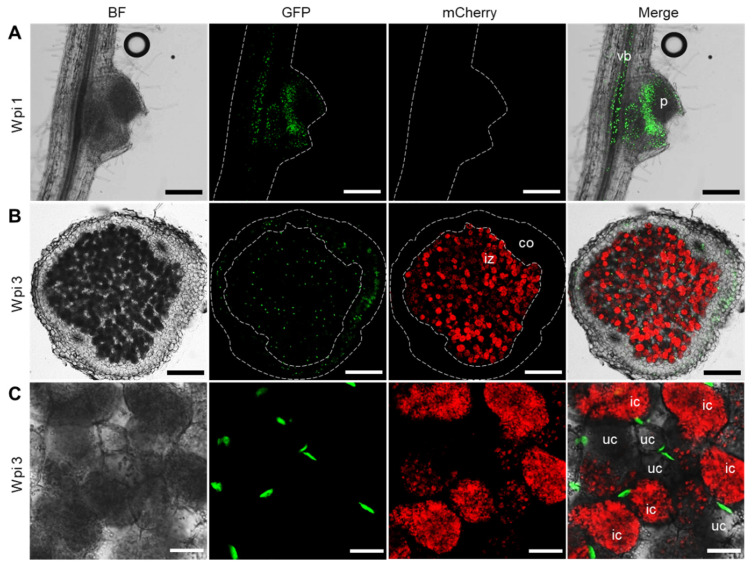
Fluorescence observation of tYFP-NLS driven by *LjβCA1* promoter in developing root nodule. Stable transgenic plants carrying the *pβCA1::tYFP-NLS* construct were inoculated with mCherry-labeled *M. loti* MAFF303099. Confocal microscopic images were captured at different developmental stages of root nodules, including young nodules at 1 wpi (**A**) and mature nodules at 3 wpi (**B**,**C**). Nuclei accumulating a green signal in the GFP channel show the fluorescence of tYFP-NLS reporter. The red signal in the mCherry channel shows the fluorescence of mCherry-labeled rhizobia. BF, bright field. Merged images of the BF, GFP, and mCherry channels were shown. vb, vascular bundle; p, primordia; iz, infected zone; co, cortical cell layers; ic, infected cell; uc, uninfected cell. Scale bars, 200 μm (**A**,**B**); 25 μm (**C**).

In contrast, we have not been able to detect any GUS staining signal in all the *pβCA1::GUS* transgenic lines. This is inconsistent with our qPCR and Western blot results, which shows an enhanced expression pattern of *LjβCA1* in symbiotic root nodules (Figure 2A,D,E). Alternatively, a tYFP-NLS reporter system was used to analyze the expression of *LjβCA1*. This reporter consists of triple YFP protein fused to nuclear localization signal peptide (NLS), which shows an accumulated fluorescent signal in nuclei. Stably transformed plants containing the *pβCA1::tYFP-NLS* construct were inoculated with *Mesorhizobium loti* MAFF303099 expressing mCherry fluorescent protein for investigating the *LjβCA1* expression pattern inside nodules. Both vascular bundle and nodule primordia show YFP fluorescent signals at 1 wpi (Figure 4A). In mature nodules, the fluorescent signals are detectable in both the inner nitrogen fixation zone and nodule cortical cell layers at 3 wpi (Figure 4B). More specifically, YFP fluorescent signals are only visible in the nuclei of uninfected cells but not in the infected cells, which are filled with mCherry-labeled rhizobia (Figure 4C). In summary, *LjαCA2*, *LjαCA6*, and *LjβCA1* exhibit quite divergent expression patterns at different types of cells, although all of them are highly expressed inside the root nodules.

### 2.4. Construction of LjCAs Mutants and Symbiotic Phenotypic Analysis

**Figure 5:**
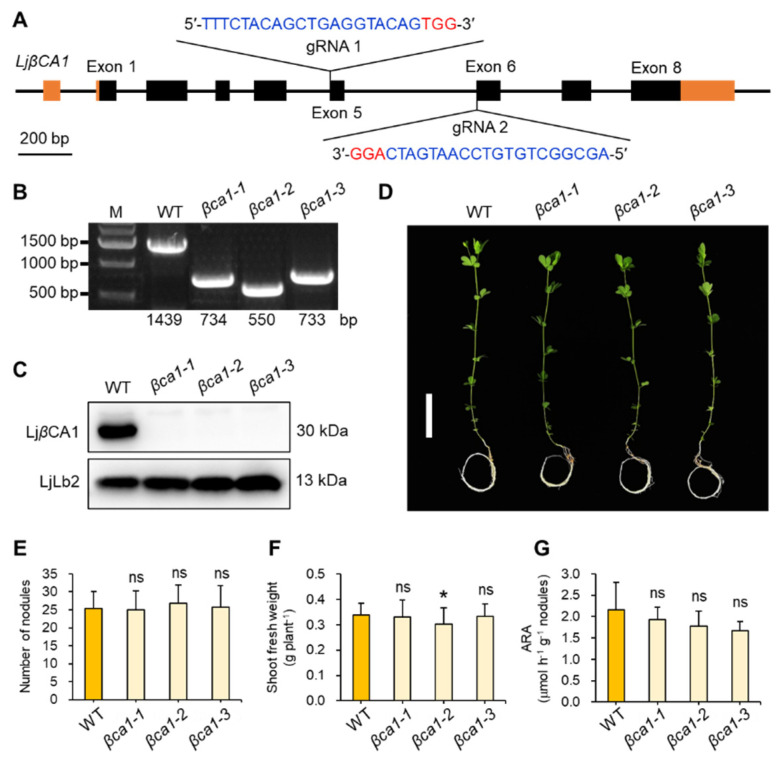
Construction and symbiotic phenotype analyses of *LjβCA1* mutants. (**A**) Gene structure and gRNA design of *LjβCA1*. Black boxes indicate the exons and orange boxes indicate the 5′ or 3′ untranslated regions (UTRs). Two gRNAs used for the *LjβCA1* knockout experiment were located in exon 5 and exon 6, respectively. The PAM sequence is marked in red. The 20 bp gRNA sequence is marked in blue. (**B**) Genotyping information of three *LjβCA1* mutants, including *βca1-1*, *βca1-2*, and *βca1-3*. (**C**) Western blot analysis of three *LjβCA1* mutants using LjβCA1 and LjLb2 antibody. (**D**) The symbiotic phenotype of *LjβCA1* mutants at 5 wpi. Plants were grown in nitrogen-deficient conditions after inoculation with *M. loti* MAFF303099. Three CRISPR/Cas9-derived independent *LjβCA1* mutant lines (*βca1-1*, *βca1-2*, and *βca1-3*) were compared to the WT plants. Scale bar, 5 cm. SNF parameters include (**E**) root nodule number, (**F**) shoot fresh weight, and (**G**) ARA per nodule fresh weight of WT and mutant plants. Values are means ± SD of 30 plants per genotype. Student’s t-test was used for statistical analysis in (**E**–**G**) by comparing respective mutant lines to WT plants. ns, not significant; *, *p* < 0.05. Phenotyping analysis has been performed three times, and similar results were obtained.

Since *LjβCA1* is the only β type CA highly expressed in root nodules, we firstly constructed the corresponding mutants of *LjβCA1* using CRISPR/Cas9 technology. As shown in Figure 5A, two gRNAs were designed against exon 5 and exon 6 of *LjβCA1*, respectively. Three independent mutant lines (*βca1-1*, *βca1-2*, *βca1-3*) were identified. All three mutant lines exhibit fragment deletion or insertion in the *LjβCA1* genomic loci, which were identified by PCR-based genotyping (Figure 5B and Figure S2). To confirm the absence of LjβCA1 protein in three mutant lines, immunoblot analysis was performed using LjβCA1 antibody. The total protein was extracted from mature nodules at 4 wpi of WT and *Ljβca1* mutants. As shown in Figure 5C, LjβCA1 protein is accumulated in wild-type mature nodules, but it is absent in the nodules of three mutant lines. In contrast, the leghemoglobin LjLb2 protein is accumulated to a similar level in mutant nodules compared with that in wild-type nodules. These results indicate that no functional LjβCA1 protein is properly translated in three independent *βca1* mutant lines.

To further investigate the biological function of LjβCA1, symbiotic phenotype analysis was performed under low nitrogen conditions (0.5 mM KNO_3_). After 5 weeks post-inoculation, three *βca1* mutants grew similarly to wild-type plants (Figure 5D). Both nodule number and shoot fresh weight showed no significant differences between WT and *βca1* mutants, except that *βca1-2* showed a slight reduction compared with WT in shoot fresh weight (Figure 5E,F). Next, the acetylene reduction activity (ARA) per nodule fresh weight was analyzed to evaluate the nitrogen fixing activity. Three *βca1* mutants showed slightly reduced ARA values, but there were no significant differences when compared with WT (Figure 5G). In conclusion, the absence of LjβCA1 does not result in obvious defects in SNF.

Considering the active expression of *LjαCA2* in the mature nodules, it was regarded as another target for gene knockout. Two common gRNAs were designed for targeting both *LjαCA1* and *LjαCA2* due to the high sequence homology between these two genes, although *LjαCA1* is a possible pseudogene (Figures S1 and S3). Two *LjαCA1/2* double mutant lines were obtained with one or two base pairs deletion in the gRNA1 targeting sequence of each gene (Figure S3). Subsequently, the symbiotic phenotype was analyzed at 4 wpi. Two double mutants (*αca12-1* and *αca12-2*) grew similarly to the wild-type plants (Figure S4A). Moreover, both nodule number and shoot biomass showed no significant difference in *αca12-1* and were slightly higher in *αca12-2* by comparison with that in wild-type plant (Figure S4B,C). Overall, knockout of both *LjαCA1* and *LjαCA2* does not influence the plant growth under normal symbiotic conditions.

## 3. Discussion

In this study, three nodule-enhanced carbonic anhydrase genes were functionally characterized in detail, which exhibited quite different expression patterns inside the root nodules of *Lotus japonicus*. Among them, *LjβCA1* and its orthologs in alfalfa (*MsCA1*) and soybean (*GmCA1*) were identified previously. Using RNA in situ hybridization and immunolocalization, the mRNA and protein of *LjβCA1*, *MsCA1*, and *GmCA1* were found to be located in most cells of nodule primordia and specific cell layers surrounding the infection zone of mature nodules [15,16,17,18,19]. In this study, the tYFP-NLS reporter system was used to confirm the promoter activity of *LjβCA1*. The tYFP-NLS expression was detected in nodule primordia and peripheral cortical cells of the mature nodules, which is consistent with the previous findings (Figure 4A,B) [15,16,17,18,19]. Interestingly, the YFP fluorescent signals were observed in uninfected cells inside the nodule infection zone, but no signal was observed in the infected cells (Figure 4C). By comparing the Western blot and qPCR results, the protein level of LjβCA1 does not decrease after nodule maturation, which is inconsistent with its reduced mRNA level after 3 wpi (Figure 2D,E). These results indicate that the LjβCA1 protein was relatively stable in the cortical and uninfected cells. However, the biological function of *LjβCA1* in these cells was still unknown.

Previous work found that another two αCA genes (*LjCAA1* and *LjCAA2*) were highly up-regulated in the root nodule of *Lotus japonicus*, which were renamed as *LjαCA2* and *LjαCA6* respectively [20] (Table 1). In this study, similar results were obtained for the expression pattern of *LjαCA2*, which is expressed in the central infection zone of mature nodules [20] (Figure 3A–D). However, different results were obtained for *LjαCA6*. The previous study has revealed that *LjαCA6* mRNA was detectable in the inner cortical cell, vascular bundle, and central tissue of nodule by using RNA in situ hybridization method [20]. Here, the promoter activity of *LjαCA6* was limited to the vascular bundle of the root and nodule using the promoter-GUS fusion system (Figure 3E–H). Two different methods obtained distinct results, which can be explained by the fact that ~3 kb of the promoter of *LjαCA6* might be not sufficient to support its native expression pattern. Notably, there is another annotated gene (*Lj5g3v0780650.1*) located around 2186 bp up-stream of *LjαCA6* translation starting site. Nevertheless, further work is needed to solve this discrepancy.

**Figure 6:**
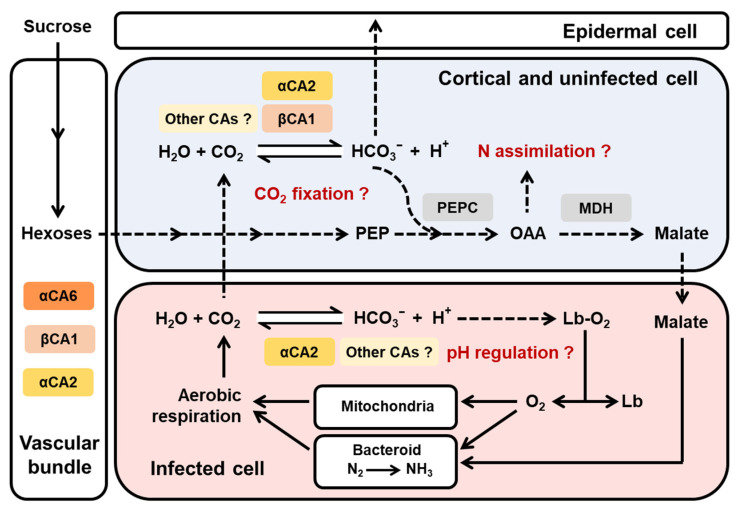
Proposed working model of carbonic anhydrases in root nodule symbiosis. Three carbonic anhydrase genes (*LjαCA2*, *LjαCA6*, and *LjβCA1*) exhibit different expression patterns inside root nodules. *LjαCA2* is expressed in both infected and uninfected cells inside the central nitrogen fixation zone. *LjαCA6* is only expressed in the vascular bundle. *LjβCA1* is expressed in the uninfected cells of the central nitrogen fixation zone and also the cortical cells around the root nodule. Other CAs have not been characterized in this work but may collaboratively play important roles in maintaining an efficient SNF by contributing to pH regulation, CO_2_ fixation, and N assimilation in various types of cells within nodules. The solid arrows indicate the verified processes, and the dashed arrows indicate the hypothetical processes awaiting further investigations. PEP, phosphoenolpyruvate; PEPC, phosphoenolpyruvate carboxylase; OAA, oxaloacetate; MDH, malate dehydrogenase; Lb, leghemoglobin.

Numerous studies have revealed the CO_2_ fixation capability of root nodules in many legume species, such as soybean, alfalfa, pea, and Lotus [23,24,25,26]. Specific genes coding for phosphoenolpyruvate carboxylase (PEPC) and malate dehydrogenase (MDH) exhibit enhanced expression in root nodules [18,27,28]. The CA-PEPC-MDH pathway was regarded as the key component of dark CO_2_ fixation in legume root nodules [29]. As shown in Figure 6, CA catalyzes the hydration of carbon dioxide, providing bicarbonate for PEPC. Oxaloacetate (OAA), the refixed organic acid molecules, can be used as the C skeleton for N assimilation and a C resource for supporting rhizobial respiration. In this model, both *LjβCA1* and *LjαCA2* are expressed in the nodule cortical and uninfected cells, which are the cell types responsible for the carbon metabolism and gas exchange barrier [16,30]. Thus, these two CAs may function in nodule CO_2_ recycling or facilitating excess CO_2_ out of the nodule (Figure 6). Additionally, LjβCA1 and its orthologs (MsCA1 and GmCA1) lack the signal peptide sequence present in AtβCA1, which is a chloroplastic carbonic anhydrase (Figure S5A). The LjβCA1-GFP fusion protein was localized in the cytoplasm in *Nicotiana benthamiana* leaves, whereas GFP itself was localized in the cytoplasm and nucleus (Figure S5B,C). Thus, LjβCA1 or other CA isoforms are probably involved in CO_2_ metabolism coupled with PEPC enzyme in the cytoplasm of the cortical and uninfected cells (Figure 6).

Another hypothetical function of CA enzymes inside symbiotic root nodules could be comparable to human red blood cells (RBCs), which contain carbonic anhydrase as the second most abundant protein next to hemoglobin [31]. CO_2_ produced by aerobic respiration can be quickly transformed into HCO_3_^−^ and H^+^ through the catalysis mediated by α-type CAs in RBCs. Once the H^+^ is combined with oxyhemoglobin, the conformation of hemoglobin changes and oxygen is released to maintain normal respiration of aerobic tissue, which is known as the Bohr effect. Consequently, α-type CAs can trigger the oxygen release from oxyhemoglobin through pH regulation [31,32,33]. In the infected cells of root nodules, a large amount of leghemoglobin associates with oxygen and creates the micro-aerobic environment, which is crucial for efficient nitrogen fixation. Leghemoglobin needs to transfer a low concentration but high-flux rate of oxygen to mitochondria and bacteroid for sustaining normal cell respiration (Figure 6). However, the mechanism in regulating the release of oxygen from oxy-leghemoglobin is still unclear [34,35,36]. Thus, we speculate that a similar regulatory mechanism might be working in the nodule infected cells as that in RBCs. In this study, *LjαCA2* showed strong induction in the infected cells of mature root nodules. LjαCA2-mediated CO_2_ rehydration may lead to the change of local pH, which influences the affinity between leghemoglobin and oxygen (Figure 6). Indeed, it has been reported that the pH value near the symbiosome membrane is significantly lower than that in other areas of the infected cells [37].

Several functions of CAs during SNF have been proposed, but none of them have been experimentally confirmed. In this study, knockout of *LjβCA1* or both *LjαCA1* and *LjαCA2* does not influence the overall plant growth under normal symbiotic conditions (Figure 5 and Figure S4). Here, explanations are given for illustrating these unexpected results. Firstly, the multiple CA isoforms may function redundantly to support efficient SNF. Indeed, these *LjCAs* exhibit partially overlapped expression patterns inside root nodules (Figure 3, Figure 4, and Figure 6). In addition, the function of *LjCAs* with low expression levels in nodules can not be ignored, such as *LjαCA5* and *LjβCA3* (Figure 2A). The other explanation is that LjCAs may help root nodules adapt to a variety of environmental stresses, such as drought, flooding, alkaline, or salinity stresses. In Arabidopsis, stress conditions can change the expression and activity of the CA enzymes. Numerous findings supported the contributions of CAs in plants adaptation to various stresses [5,38]. Therefore, the phenotype of *LjCAs* mutants would be more obvious under specific stress conditions but is not shown under the normal symbiotic conditions. Future work will focus on the construction of multi-gene knockout mutants of *LjCAs* to decipher the functional redundancy of carbonic anhydrases inside root nodules. The symbiotic phenotype analysis of *LjCAs* mutants under different stress conditions could be systemically performed. Finally, the characterization of subcellular localizations of LjCAs in different cell types of root nodules would also provide valuable information regarding how an efficient SNF is systemically coordinated in mature nodules.

## 4. Materials and Methods

### 4.1. Gene Identification and Phylogenetic Analysis

The protein sequences of AtCAs were obtained from the TAIR database (http://www.arabidopsis.org) (accessed on 19 September 2018). To identify the CA-encoding genes in the *Lotus japonicus* genome, we performed a BLASTP search on the Kazusa DNA Research Institute website (http://www.kazusa.or.jp/lotus/) (accessed on 19 September 2018), using the known AtCAs as queries with the parameters id% > 50% and E-value < 10^−15^. Then, the protein sequences were analyzed in the NCBI conserved domain database with default parameters (https://www.ncbi.nlm.nih.gov/Structure/cdd/wrpsb.cgi) (accessed on 30 May 2020). The protein biochemical properties such as the theoretical isoelectric point (pI), the molecular weight (MW), and the grand average of hydropathicity (GRAVY) were calculated using the ProtParam program (https://web.expasy.org/protparam/) (accessed on 4 March 2021). Multiple sequence alignment of the AtCAs and LjCAs was performed using Clustal W. Subsequently, a phylogenetic tree was constructed using MEGA 7.0 with the neighbor-joining method (1000 bootstrap replications) [39].

### 4.2. Plant Growth and Transformation

Ecotype MG-20 of *Lotus japonicus* was used in all experiments [40]. MG-20 seeds were firstly treated with 98% sulfuric acid for 10 min, subsequently washed three times with sterile water, then surface-sterilized in 2% sodium hypochlorite for 5 min, and washed three times again. The sterilized seeds were kept at 4 °C for at least 24 h. Then, seeds were germinated on one-half strength Murashige–Skoog medium in the dark for 2 days at 24 °C, which was followed by illumination for another 3–4 days. The stable transformation was performed as previously described [41]. For inoculation assay, five-day-old seedlings were planted in pots containing sterile perlite:vermiculite (1:3) supplemented with B&D medium containing 0.5 mM KNO_3_ [42] and grown in a greenhouse at 24 °C under 16 h/8 h day/night cycle. Ten-day-old seedlings were inoculated with *Mesorhizobium loti* MAFF303099, wild-type, or mCherry-labeled strain [43,44]. For subcellular localization experiments, *Nicotiana benthamiana* seedlings were grown in pots filled with perlite:vermiculite:nutrient soil mixture (1:1:1). The one-month-old tobacco plants were used for infiltration with *Agrobacterium tumefaciens* EHA105.

### 4.3. GUS Staining and tYFP-NLS Observation

Promoter fragments (~3 kb) of *LjαCA2*, *LjαCA6*, and *LjβCA1* were PCR amplified using MG-20 genomic DNA as a template and then cloned into the promoterless DX2181G vector. For *LjβCA1*, another construct (pC1300-tYFP-NLS) was used to indicate the promoter activity. Related primers are included in Table S2. Stable transgenic plants were used for GUS staining and fluorescence observation. At least eight independent transgenic lines were tested. For GUS staining, plant tissues were immersed in the staining buffer containing 0.5 mg/mL of 5-bromo-4-chloro-3-indolyl-β-D-glucuronic acid cyclohexylammonium salt (Sangon Biotech, Shanghai, China), 1 mM potassium ferricyanide, 1 mM potassium ferrocyanide, 100 mM potassium phosphate (pH 7.0), 10 mM EDTA, 0.1% (*w*/*v*) sodium lauroyl sarcosinate, and 0.1% (*v*/*v*) Triton X-100. The reaction was performed at room temperature in a vacuum overnight. If necessary, root nodules were sectioned to 50–80 μm thick slices using a vibratome (Leica VT 1000S, Nussloch, Germany). Images were captured with a fluorescence stereo microscope (Nikon SNZ18, Tokyo, Japan) for the whole nodule and a light microscope (Leica DM2500, Nussloch, Germany) for nodule sections. For fluorescence observation, nodules were sectioned to 80–100 μm slices first and then observed and photographed with a laser-scanning confocal microscope (Leica TCS SP8, Nussloch, Germany). Fluorescence was detected with excitation at 488 nm and emission at 500–550 nm for tYFP-NLS and GFP, excitation at 580 nm and emission at 560–630 nm for mCherry, and excitation at 488 nm and emission at 650–750 nm for chloroplast autofluorescence.

### 4.4. Construction of LjCAs Knockout Mutants

Using CRISPR/Cas9 technology, *LjCAs* mutants were obtained through stable transformation in a MG-20 background. The web tool CRISPR-P 2.0 was used for designing high-score guide RNAs with low off-target effect (http://cbi.hzau.edu.cn/crispr/) (accessed on 27 March 2016). Two guide RNAs were designed and cloned into the final CRISPR vector as described before [45]. PCR-sequencing-based genotyping was performed at T_1_ generation. Cas9-free and homozygous mutants were identified and allowed to self-cross to produce T_2_ seeds. The symbiotic phenotype was analyzed using at least T_2_ generation plants. The detailed procedure for CRISPR-Cas9 mediated genome editing in *Lotus japonicus* was described previously [46]. The genotypes of obtained *LjCAs* mutants are described in Figures S2 and S3. Related primers were included in Table S2.

### 4.5. RNA Extraction and qRT-PCR

Total RNA was isolated using TransZol Plant reagent (TransGen Biotech, Beijing, China). 1 µg total RNA was used to synthesize the first-strand cDNA according to the instructions of the HiScript II Q RT SuperMix (Vazyme Biotech, Nanjing, China). Real-Time qRT-PCR was performed on Bio-Rad CFX96 Real-Time PCR Detection System based on the instruction of the TransStart^®^ Tip Green qPCR SuperMix (TransGen Biotech, Beijing, China). The ubiquitin gene (GenBank accession no. AW720576) served as a reference gene. All reactions were performed with three technical replications. Primers used in this study were included in Table S2.

### 4.6. Western Blot Analysis

Root and nodule tissues (~200 mg) were homogenized in liquid nitrogen. Total protein was precipitated with methanol–chloroform and dissolved in 50 mM Tris-HCl, pH 6.8, 2% (*w*/*v*) SDS [47]. Protein concentration was quantified by BCA protein assay (Sangon Biotech, Shanghai, China). Similar amounts of total protein (~30 µg) were separated on 10% SDS-PAGE gel and transferred to the PVDF membrane (Merck Millipore, Darmstadt, Germany). Immunoblot analysis was performed using a primary antibody raised against LjLb2, LjβCA1, and NifK. To generate these antibodies, full-length coding sequences of LjβCA1, LjLb2, and MlNifK were amplified and cloned into the pET-28a vector. The sequence-verified constructs were transformed into *E. coli* BL21 (DE3)-RIL strain. The recombinant proteins with an N-terminal 6xHis tag were expressed under 28°C with 0.5 mM IPTG and then purified by Ni-NTA affinity chromatography (Genscript, Nanjing, China). The purified recombinant proteins were used as antigens for obtaining the primary antibodies in rabbit (PHYTOAB, San Jose, CA, USA). HRP-conjugated goat anti-rabbit secondary antibody was used. Signals were detected with Western ECL Substrate (Bio-Rad, Hercules, CA, USA) under a ChemiScope western blot processor (ChemiScope 6300, Clinx Science Instruments, Shanghai, China).

### 4.7. Nitrogenase Activity Assay

Nitrogenase activity was measured using the acetylene reduction assay [48]. Nodulated roots were put into glass bottles sealed with rubber stoppers. Each bottle contained four nodulated roots, and 5 biological replicates of each genotype were analyzed. 2 mL of acetylene was injected into each bottle after the same volume of air was pumped out. Subsequently, all bottles were incubated for 2 h at 28°C. For each bottle, a 100 μL gas sample was used to measure the ethylene production using a GC-4000A gas chromatograph (East & West Analytical Instruments, Beijing, China).

## Figures and Tables

**Table 1:** Features of the 13 CA proteins (LjCAs) identified in *Lotus japonicus*.

Type	Gene	Chr.	Transcript ID	Former Name	NO. of AA	MW (kDa)	pI	GRAVY
α type	*LjαCA1*		Lj0g3v0129349.1		269	30.47	8.79	−0.454
	*LjαCA2*	Chr 1	Lj1g3v4226880.1	*LjCAA1* [20]	269	30.29	9.05	−0.453
	*LjαCA3*	Chr 3	Lj3g3v3082370.1		218	24.65	5.92	−0.456
	*LjαCA4*	Chr 5	Lj5g3v0670150.1		280	32.01	9.66	−0.619
	*LjαCA5*	Chr 5	Lj5g3v0670540.1		266	30.61	6.95	−0.639
	*LjαCA6*	Chr 5	Lj5g3v0780660.1	*LjCAA2* [20]	274	30.74	6.63	−0.374
β type	*LjβCA1*	Chr 1	Lj1g3v0410090.1	*LjCA1* [17]	263	29.87	6.00	−0.303
	*LjβCA2*	Chr 2	Lj2g3v1002750.2		324	34.90	6.54	−0.058
	*LjβCA3*	Chr 2	Lj2g3v1403790.1		256	27.91	5.49	−0.129
	*LjβCA4*	Chr 6	Lj6g3v2193530.1		263	29.05	6.44	−0.186
γ type	*LjγCA1*	Chr 1	Lj1g3v2124850.1		273	29.58	6.23	−0.095
	*LjγCA2*	Chr 2	Lj2g3v1731290.1		271	29.46	6.07	−0.101
	*LjγCAL1*	Chr 4	Lj4g3v2916460.1		186	20.20	9.44	0.176

Chr, chromosome; NO. of AA, number of amino acids; pI, isoelectric point; MW, molecular weight; GRAVY, grand average of hydropathicity.

## Supplementary Materials

The following are available online at https://www.mdpi.com/article/10.3390/ijms22157766/s1.

## Abbreviations

ARA	Acetylene reduction activity
BCA	Bicinchoninic acid
CA	Carbonic anhydrase
CCM	CO_2_ concentrating mechanism
CRISPR	Clustered regularly interspaced short palindromic repeats
EDTA	Ethylenediaminetetraacetic acid
GRAVY	Grand average of hydropathicity
GUS	β-Glucuronidase
Lb	Leghemoglobin
MDH	Malate dehydrogenase
MW	Molecular weight
NLS	Nuclear localization signal
OAA	Oxaloacetate
PEP	Phosphoenolpyruvate
PEPC	Phosphoenolpyruvate carboxylase
pI	Isoelectric point
PVDF	Polyvinylidene difluoride
RBCs	Red blood cells
SDS	Sodium dodecyl sulfate
SNF	Symbiotic nitrogen fixation
tYFP	Triple yellow fluorescent protein
