# Supplementary material for: Molecular Characterization of Carbonic Anhydrase Genes in Lotus japonicus and Their Potential Roles in Symbiotic Nitrogen Fixation
Source: Int J Mol Sci. 2021 Jul 21;22(15):7766. doi: 10.3390/ijms22157766 (PMC8346106; doi:10.3390/ijms22157766)
Supplement: Supplementary file 1 [file ijms-22-07766-s001.zip › ijms-1283974-supplementary.pdf]

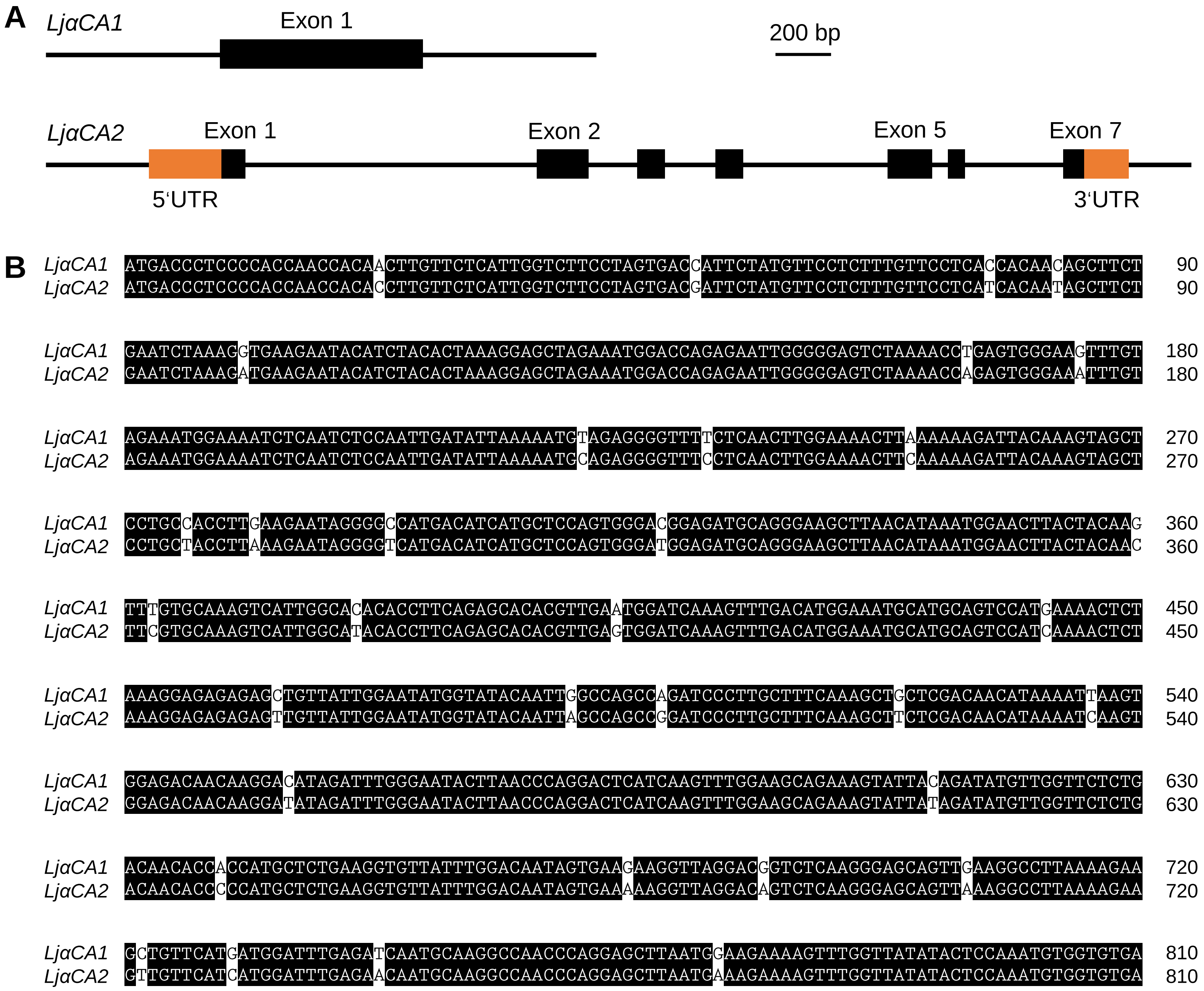

Figure S1. Gene models and coding sequence alignment of *LjαCA1* and *LjαCA2*. (A) Gene models of *LjαCA1* and *LjαCA2*. Black boxes indicate the exons and orange boxes indicate the 5’ or 3’ untranslated regions (UTRs). (B) Coding sequence alignment of *LjαCA1* and *LjαCA2*. Sequence alignment was performed using DNAMAN software with default parameters.

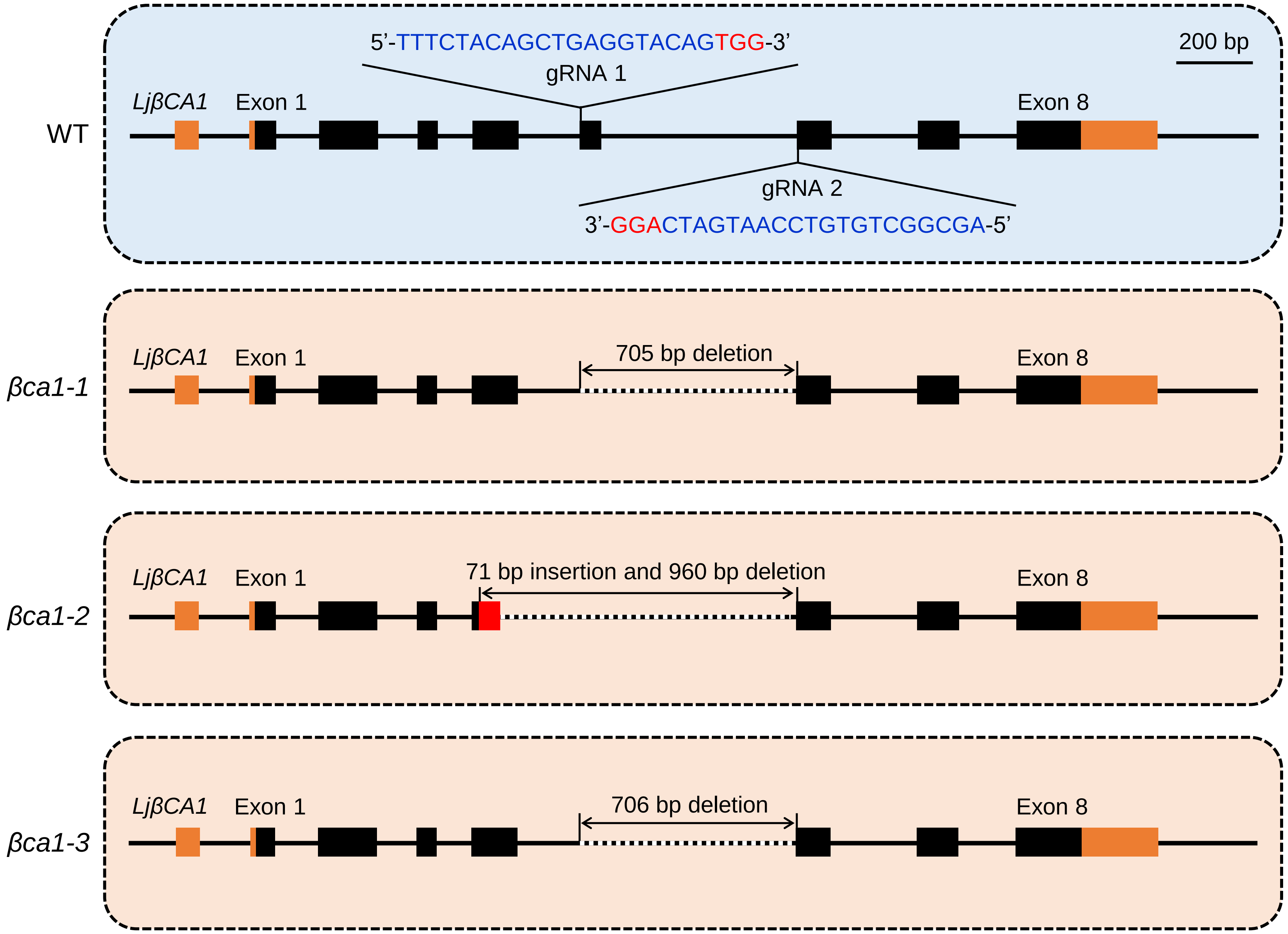

Figure S2. Genotyping analysis of CRISPR/Cas9-derived *LjβCA1* mutants. The sequences of *LjβCA1* in each mutant line were confirmed by PCR and sequencing. The schematics show the genotyping information of *LjβCA1* in *βca1-1*, *βca1-2*, *βca1-3* mutants. Black boxes indicate the exons and orange boxes indicate the 5' or 3' untranslated regions (UTRs). Two gRNAs used for the *LjβCA1* knockout experiment were located in exon 5 and exon 6 respectively. PAM sequence was marked in red. 20 bp gRNA sequence was marked in blue. Dotted lines indicate the fragment deletion. The red box indicates the fragment insertion.

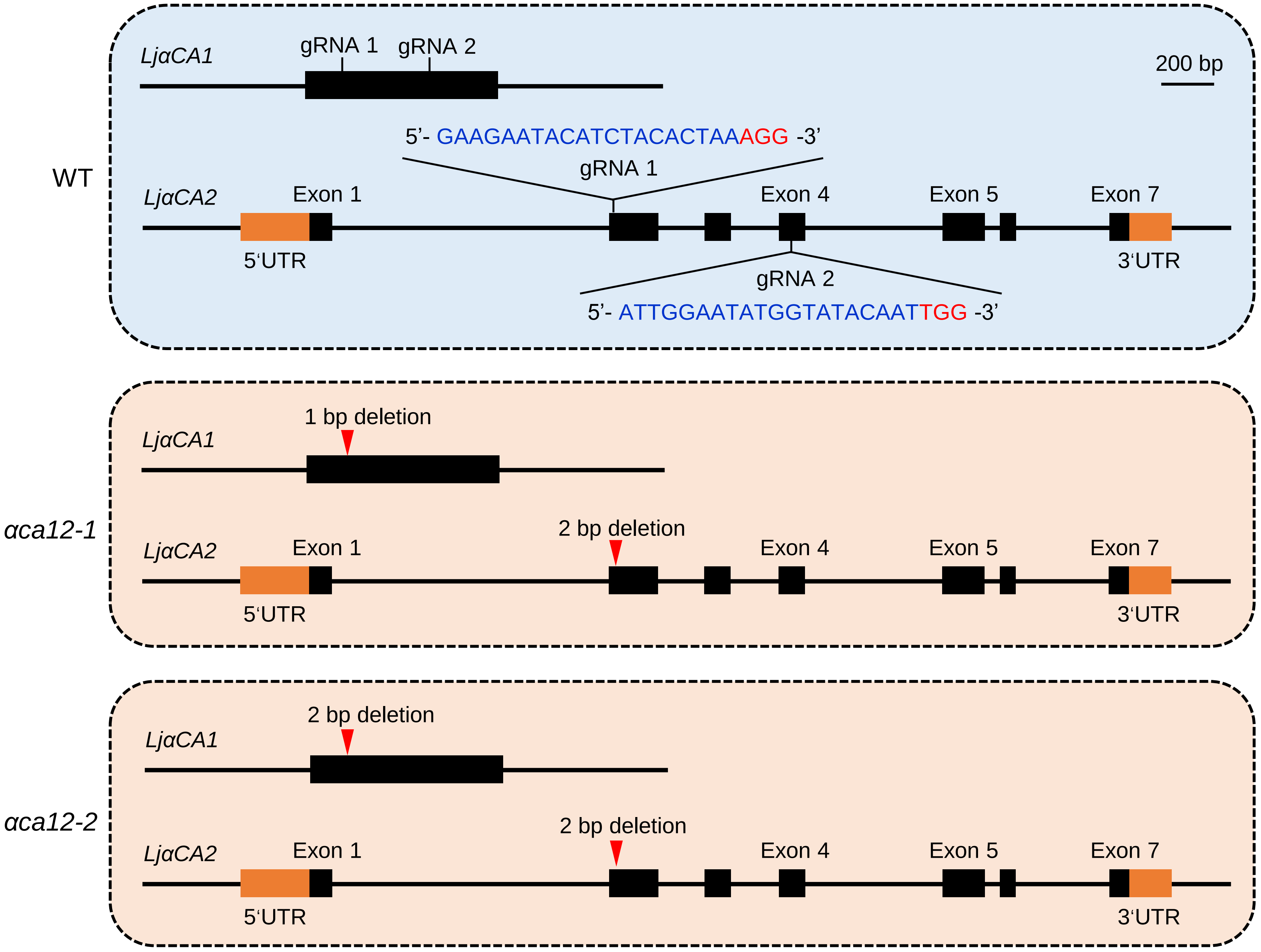

Figure S3. Genotyping analysis of CRISPR/Cas9-derived *LjαCA1/2* double mutants. The sequences of *LjαCA1* and *LjαCA2* in each double mutant line were confirmed by PCR and sequencing. The schematics show the genotyping information of *LjαCA1* and *LjαCA2* in *αca12-1*, *αca12-2* mutants. Black boxes indicate the exons and orange boxes indicate the 5' or 3' untranslated regions (UTRs). Two gRNAs used for *LjαCA2* knockout experiment were located in exon 2 and exon 4 respectively. PAM sequence was marked in red. 20 bp gRNA sequence was marked in blue. Red triangles indicate the deletion with 1 or 2 base pairs.

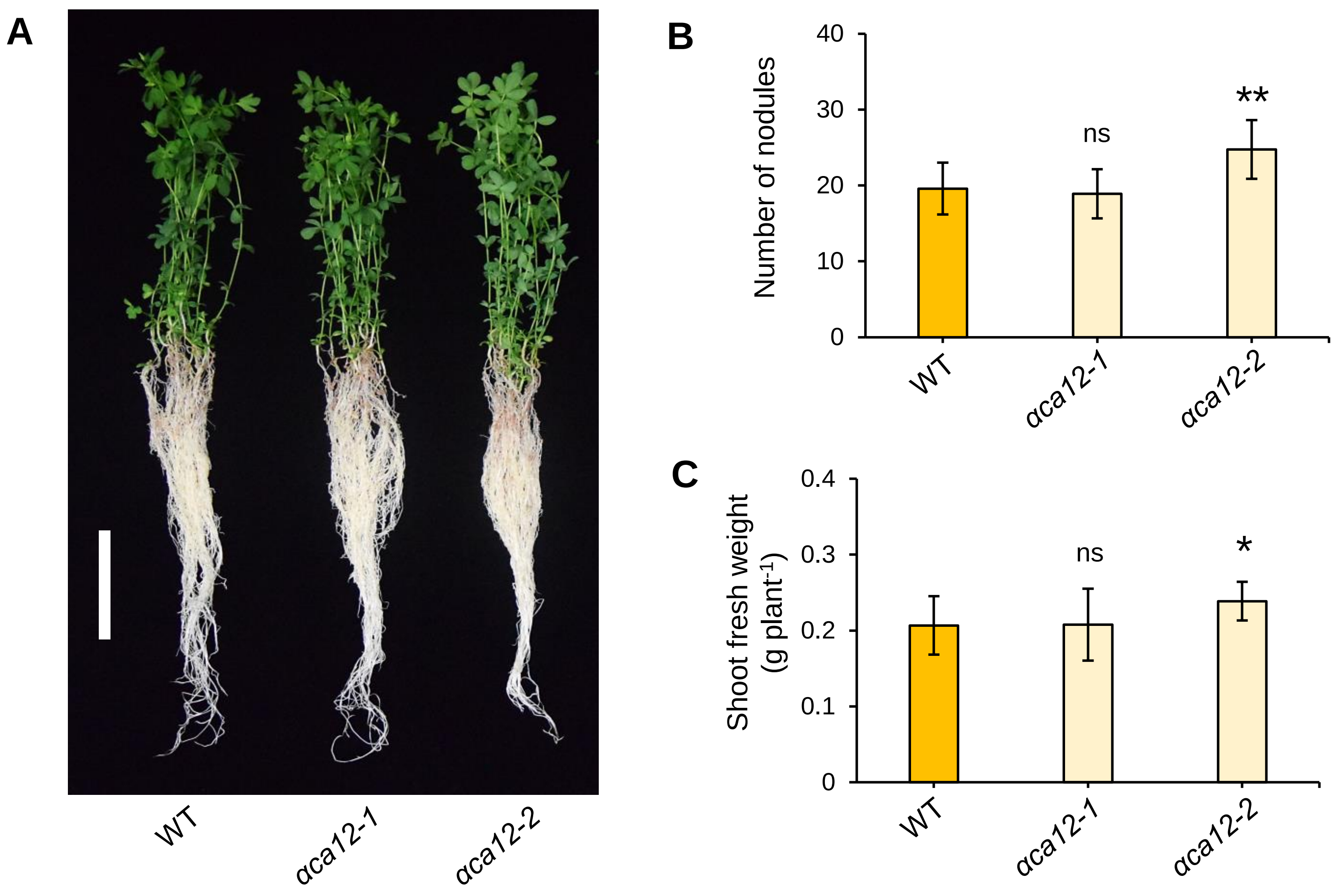

Figure S4. Symbiotic phenotypes of *LjαCA1* and *LjαCA2* mutants. (A) Symbiotic phenotypes of plants at 4 wpi. Plants were grown in nitrogen-deficient conditions after inoculation with *M. loti* MAFF303099. Two CRISPR/Cas9-derived mutants (*αca12-1* and *αca12-2*) were compared to the WT plants. Scale bar, 5 cm. (B) Root nodule number, (C) Shoot fresh weight of WT and mutant plants. Values are means ± SD of 12 plants per genotype. Student's t-test was used for statistical analysis in (B and C). ns, not significant; *, $P < 0.05$; **, $P < 0.01$. Phenotyping analysis has been performed three times and similar results were obtained.

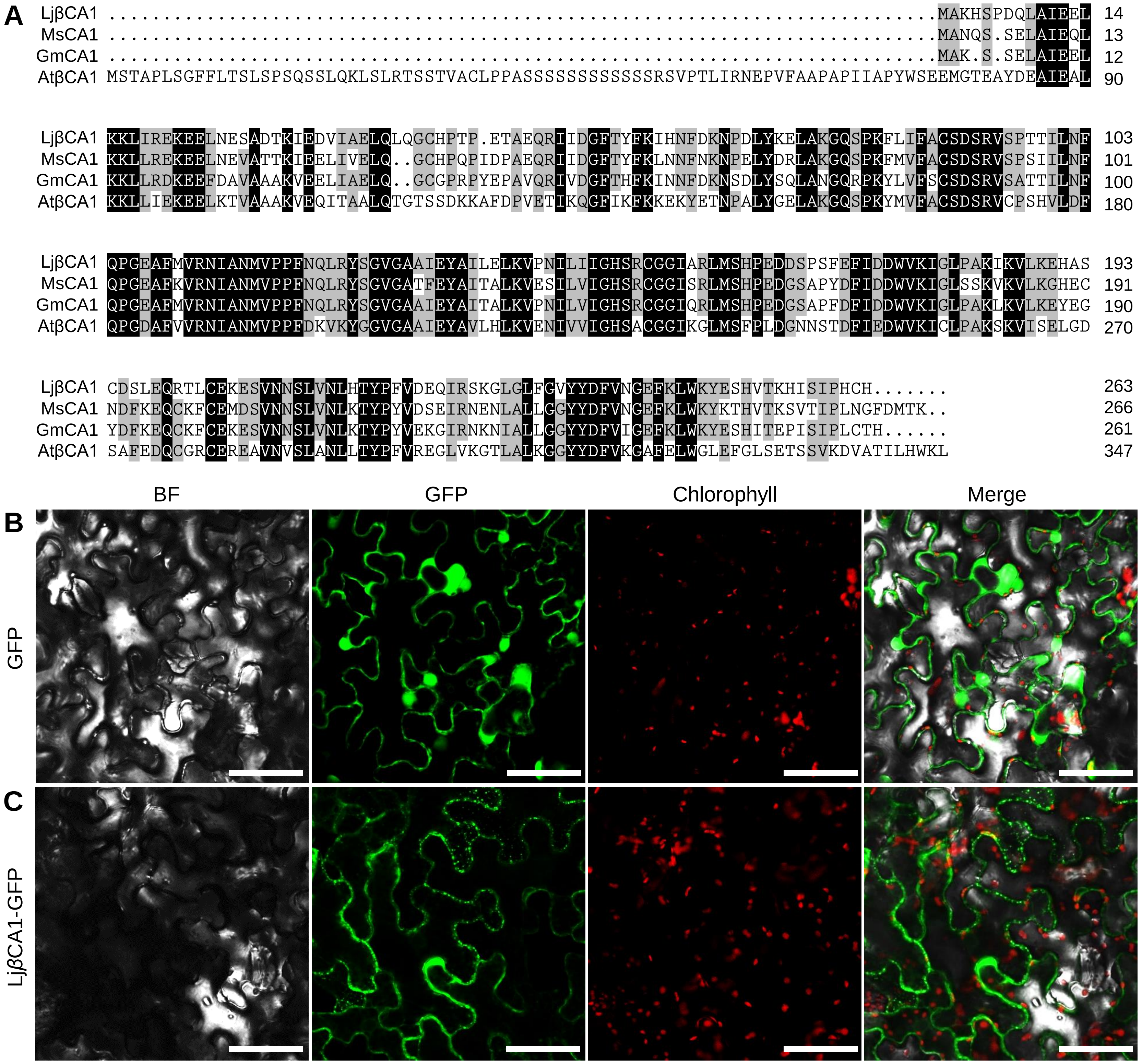

Figure S5. Subcellular localization of LjβCA1 in *Nicotiana benthamiana* leaves. (A) Protein sequence alignment of LjβCA1 and its homologs in Alfalfa, Soybean, and Arabidopsis. LjβCA1 (Lj1g3v0410090.1), MsCA1 (GenBank: CAA63712.1), GmCA1 (GenBank: CAB43571.1), and AtβCA1 (At3g01500.2). Sequence alignment was performed using DNAMAN software with default parameters. (B-C) Tobacco epidermal cells were imaged at 48 hrs after infiltration for GFP (B) or LjβCA1-GFP fusion protein (C). The green signal in the GFP channel shows fluorescence from GFP or LjβCA1-GFP fusion protein. The red signal in the Chlorophyll channel shows the autofluorescence of chlorophyll. BF, bright field. Merged images of the BF, GFP, and Chlorophyll channels were shown. Scale bars, 50 μm (B, C).

Supplemental Table 1. Expression profiles of *LjCAs* in different plant tissues in *Lotus japonicus* Expression Atlas

Gene	Gene ID	Root	Stem	Leaf	Flower	Pod-20d	Seed-20d	Nodule-21d
LjαCA1	Lj0g3v0129349	17	18	16	25	18	17	2908
LjαCA2	Lj1g3v4226880	18	19	15	123	17	16	10419
LjαCA3	Lj3g3v3082370	18	16	13	13	15	13	22
LjαCA4	Lj5g3v0670150	304	99	43	149	31	41	157
LjαCA5	Lj5g3v0670540	-	-	-	-	-	-	-
LjαCA6	Lj5g3v0780660	17	694	235	423	61	24	4693
LjβCA1	Lj1g3v0410090	17	15	18	15	13	13	14023
LjβCA2	Lj2g3v1002750	150	9414	18082	7371	526	32	63
LjβCA3	Lj2g3v1403790	6563	2795	3634	2192	3418	1289	2635
LjβCA4	Lj6g3v2193530	278	918	1823	1203	486	434	131
LjγCA1	Lj1g3v2124850	5197	3376	2823	3645	3106	3198	5372
LjγCA2	Lj2g3v1731290	2637	2349	2285	2655	2725	2895	3154
LjγCAL1	Lj4g3v2916460	2852	2505	1537	2015	1517	1794	2451

These data were retrieved from *Lotus japonicus* Expression Atlas (https://lotus.au.dk/expat/). Raw data of expression levels of *LjCA*s were shown in this table.

Supplemental Table 2. Primers used for plasmid construction, RT-PCR and genotyping

**Oligo Name**	**Sequence (5'->3')**	**Purpose**
αCA2pro2934-F	GATCTACAGCGCTGACGCTCGGAGTATGATCTAGATG	Construct *pαCA2::GUS*
αCA2pro-R	ACTGACCACCCGGGGGTGGTGGTGTGACTATTGATC	Construct *pαCA2::GUS*
αCA6pro3002-F	GATCTACAGCGCTGATGTATGTCTTGGTGCACTGTG	Construct *pαCA6::GUS*
αCA6pro-R	ACTGACCACCCGGGGGTGGATAGCTAGCTGTGTATG	Construct *pαCA6::GUS*
βCA1pro2764-F	GATCTACAGCGCTCACGGAGATCCCAAATAAGTGGTG	Construct *pβCA1::GUS*
βCA1pro-R	ACTGACCACCCGGGATTTTTCCCTTTCTTCTGCAG	Construct *pβCA1::GUS*
βCA1pro-tYFP-F	AGATCTACAGCGCTAGAGATCCCAAATAAGTGGTG	Construct *pβCA1::tYFP-NLS*
βCA1pro-tYFP-R	GTCGACCTGCAGCCAATTTTTCCCTTTCTTCTGCAG	Construct *pβCA1::tYFP-NLS*
F-BamHI-βCA1-CDS	AGTGGATCCATGGCAAAGCATTCACCTGAC	Construct *p35S::βCA1-GFP*
R-SmaI-βCA1-CDS	CACCCCGGGGTGACAGTGAGGGATAGAAATG	Construct *p35S::βCA1-GFP*
F-RsaI-βCA1-gRNA1	GTTCGTTTCTACAGCTGAGGTACAG	Construct *βCA1* CRISPR plasmid
R-RsaI-βCA1-gRNA1	AAACCTGTACCTCAGCTGTAGAAAC	Construct *βCA1* CRISPR plasmid
F-BclI-βCA1-gRNA2	GTTCGAGCGGCTGTGTCCAATGATC	Construct *βCA1* CRISPR plasmid
R-BclI-βCA1-gRNA2	AAACGATCATTGGACACAGCCGCTC	Construct *βCA1* CRISPR plasmid
βCA1-exon3-F	ACCCGGATCTATACAAGGAAC	Genotyping of *βca1* mutants
βCA1-exon7-R	CACCCAATCGTCTATGAACTC	Genotyping of *βca1* mutants
βCA1-intron5-R	TGAAATGGGTGAGTTGTCTG	Genotyping of *βca1* mutants
βCA1-intron4-F	TCTGCAGCATCATCACTCTG	Genotyping of *βca1* mutants

βCA1-exon6-R	CGTCTTACAAGGAAGGAGAATC	Genotyping of *βca1* mutants
F-αCA12-gRNA1	GTTCGAAGAATACATCTACACTAA	Construct *αCA1/2* CRISPR plasmid
R-αCA12-gRNA1	AAACTTAGTGTAGATGTATTCTTC	Construct *αCA1/2* CRISPR plasmid
F-αCA12-gRNA2	GTTCGATTGGAATATGGTATACAAT	Construct *αCA1/2* CRISPR plasmid
R-αCA12-gRNA2	AAACATTGTATACCATATTCCAATC	Construct *αCA1/2* CRISPR plasmid
αCA1-intron1-F	CTGCACCAATGAGATGACAG	Genotyping of *αCA1/2* mutants
αCA1-intron4-R	AACTCAACCACACCAGCATG	Genotyping of *αCA1/2* mutants
αCA2-CDS-F	AGTGGATCCATGACCCTCCCCACCAACCAC	Genotyping of *αCA1/2* mutants
αCA2-CDS-R	CACCCCGGGCACCACATTTGGAGTATATAAC	Genotyping of *αCA1/2* mutants
Ub-SqF	CGTGAAGGCTAAGATCCAGGATAAG	Semi-quantitative RT-PCR
Ub-SqR	CGATACTACTTGTTCAAGAGGGGC	Semi-quantitative RT-PCR
βCA2-SqF	CAGCTAGGGACAACATCATC	Semi-quantitative RT-PCR
βCA2-SqR	TGTGCAGAGCTCTCCAAAAG	Semi-quantitative RT-PCR
βCA3-SqF	ATTGGACATAGCTGCTGTGG	Semi-quantitative RT-PCR
βCA3-SqR	CCACAGCTCAAAATTGCCAG	Semi-quantitative RT-PCR
βCA4-SqF	GCTGAAGCTGAAGATGAGTGTG	Semi-quantitative RT-PCR
βCA4-SqR	GCATTTGTTTCTGTGGGTCC	Semi-quantitative RT-PCR
αCA3-SqF	CCATTGGCATTCTCCATCTG	Semi-quantitative RT-PCR
αCA3-SqR	GGTCTTGCATTTGTATCCGAG	Semi-quantitative RT-PCR
αCA4-SqF	CACTCACCTTCTGAACACAC	Semi-quantitative RT-PCR

αCA4-SqR	CTCTGAGCATGATCATGGAC	Semi-quantitative RT-PCR
αCA5-SqF	GGCAACAAGATAGCTGTGGT	Semi-quantitative RT-PCR
αCA5-SqR	CTTGGCCTTGCATTCATCTC	Semi-quantitative RT-PCR
Ubi-qF	TTCACCTTGTGCTCCGTCTTC	Quantitative RT-PCR
Ubi-qR	AACAACAGCACACACAGACAATC	Quantitative RT-PCR
βCA1-qF	CCAAACATCCTGATCATTGGAC	Semi-quantitative and quantitative RT-PCR
βCA1-qR	CTTGATTTTGGCAGGTAAACCA	Semi-quantitative and quantitative RT-PCR
αCA2-qF	TGAGATCAATGCAAGGCCAAC	Semi-quantitative and quantitative RT-PCR
αCA2-qR	CCCTTCACACCACATTTGGAGT	Semi-quantitative and quantitative RT-PCR
αCA6-qF	ATGCCCAAATTCCTATCGGC	Semi-quantitative and quantitative RT-PCR
αCA6-qR	CCTGCTGTGTGTCCTTGCATT	Semi-quantitative and quantitative RT-PCR
